# Neutrophil to lymphocyte ratio and fibrinogen values in predicting patients with type B aortic dissection

**DOI:** 10.1038/s41598-021-90811-5

**Published:** 2021-05-31

**Authors:** Shuangshuang Li, Jin Yang, Jian Dong, Renle Guo, Sheng Chang, Hongqiao Zhu, Zhaohui Li, Jian Zhou, Zaiping Jing

**Affiliations:** 1 Department of Vascular Surgery, Changhai Hospital, Navy Medical University, Shanghai, 200433 China; 2 Department of Vascular Surgery, Suining Central Hospital, No. 127 Desheng West Road, Chuanshan District, Suining, 629000 Sichuan Province China; 3 Department of Vascular of Surgery, The First People’s Hostpital of Yibin, Yibin, 644000 China

**Keywords:** Biomarkers, Diseases

## Abstract

The aim of this study is to detect the diagnosis value of neutrophil lymphocyte ratio (NLR) and fibrinogen (FIB) in type B aortic dissection (TBAD) patients. This retrospective observation study consisted patients with TBAD, aortic aneurysm and physical examination between January 1, 2016 and December 31, 2019. Demographic and clinical information after the first admission were collected. Multivariate logistic regression analysis was performed to explore the correlational relationship between NLR, FIB and TBAD. Receiver Operating Characteristic Curve (ROC) was performed to evaluate the diagnostic implication of NLR and FIB in TBAD patients. Six hundred and six patients who were first diagnosed with TBAD were included. Control groups were 202 aortic aneurysm and 140 physical examination subjects. The level of NLR and FIB in aortic dissection patients was significantly higher than aortic aneurysm patients and healthy group (P < 0.001). According to the results of multivariate logistic regression analysis, NLR and FIB were independent risk factors of aortic dissection, and the odds ratio (OR) and 95% confidence interval (CI) value of NLR and FIB were 1.499 (1.126–1.738) and 1.914 (1.475–2.485), respectively. The area under the curve (AUC) was 0.836 of NLR and 0.756 of FIB. NLR and FIB showed high specificity, 89% and 83% respectively. This is the first study provided information on the diagnosis performance of NLR and FIB in TBAD patients. NLR and FIB showed high specificity, which may be a valuable tool for the diagnosis of TBAD.

## Introduction

Aortic dissection (AD) is the most dangerous vascular disease that requires early recognition to achieve better clinical management^1^. AD was classified as type A and type B according to Stanford criteria^2,3^. Stanford type B AD (TBAD) accounted for 25% to 40% of all aortic dissections^4^. TBAD lacks of typical symptoms and always share similar clinical presentations with aortic aneurysm, myocardial infarction or pulmonary embolism^5^. As to auxiliary examination, the electrocardiogram (ECG) and chest x-ray lacks of sensitivity and specificity for it. Computed tomography angiography (CTA) and magnetic resonance angiography (MRA) need long appointment time. Diagnosis is therefore time consuming, as a result, a large fraction of patients in whom the diagnosis may be mistaken or overlooked^6–9^.

Biomarkers can provide valuable information to help distinguishing aortic dissection patients. Preliminary evidence suggested a possible diagnostic role of D-dimer, Smooth muscle myosin and so on^10–12^. However, these biomarkers were still not yet routinely used in clinical practice, which indicated that available biomarkers were not enough for recognition and management of aortic dissection. Therefore, and additional useful biomarkers are urgently needed. Inflammation played a key role in AD initiate and progression^13,14^. AD involves different inflammatory mediators, which act alone or together to cause damage to the blood vessel wall. For example, S100A12 is a pro-inflammatory protein specific to early recruited phagocytes, which can enhance the migration of activated monocytes to the blood vessel wall, plays a key role in systemic inflammation^15^, and may be a promising biomarker for predicting the occurrence and prognosis of AD^16^. IL-22 is an inflammatory mediator, which can affect the process of AD by regulating vascular smooth muscle contraction and macrophage infiltration^17,18^. Macrophages can release metalloproteinases and pro-inflammatory cytokines to cause extracellular matrix degradation and trigger AD, in which MMP-12 is considered to be a specific marker of aortic wall disease^19,20^. In addition, the continuous increase of C-reactive protein, IL-6 and TNF- α is also related to the development of AD^21^.

Neutrophil lymphocyte ratio (NLR) and Fibrinogen (FIB) have been found to be higher in clinical practice. They has been assessed as a valuable diagnostic biomarker involved in the process of coagulation and hemostasis and different in type A aortic dissection (TAAD)^22–24^. The role of NLR and FIB in predictor of type B aortic dissection (TBAD) is unknown. In this study, data of plasma NLR and FIB in TBAD patients, aneurysm patients, and health people was collected to evaluate its diagnostic performance.

## Methods

### Study population

**Figure 1:**
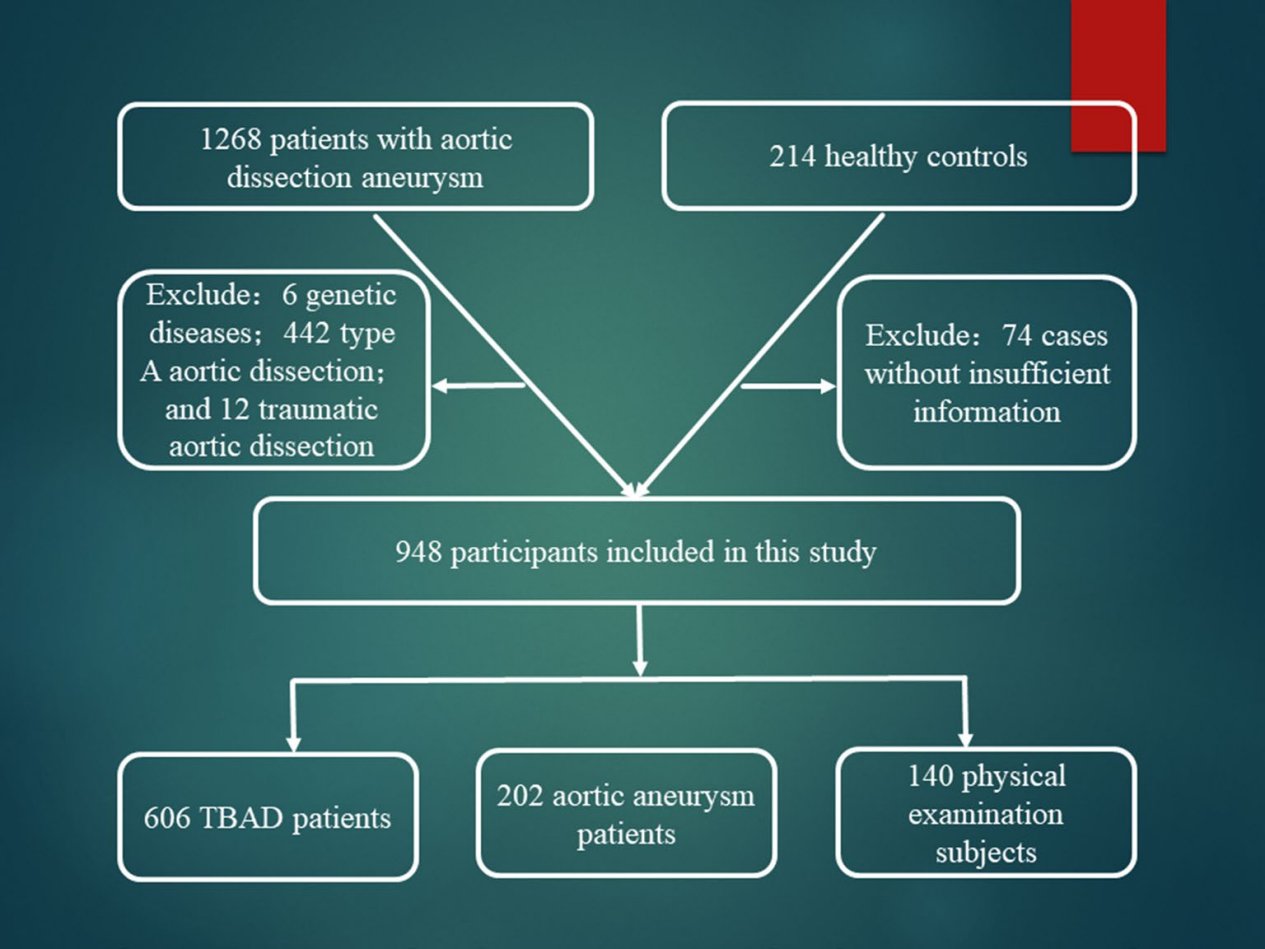
Flowchart of inclusion of the patients.

TBAD and aortic aneurysm was diagnosed by clinician according to physical examination and image examination (CTA or MRA). Demographic and clinical variables were retrospectively collected from medical charts and electronic medical records (code as 171.001—aortic dissection aneurysm). Then 1268 aortic dissection aneurysm and 214 healthy controls were retrieved between January, 1, 2016 and December 31, 2019. After screening, 442 type A aortic dissection, 12 traumatic aortic dissection, 6 genetic diseases patients, and 74 cases without insufficient information were excluded. Finally, 948 participants were included in this study, 606 TBAD patients, 202 aortic aneurysm and 140 physical examination subjects. Then we named the three cohorts as dissection group, aneurysm group and healthy group, respectively. Flowchart of inclusion of the patients can been seen in Fig. 1. All methods in this study were carried out in accordance with the guidelines Reporting of Observational Studies in Epidemiology (STROBE). The Written consent was obtained from all the participants and the study was approved by the Changhai Hospital medical ethics committee.

### Data collection

Blood samples were obtained immediately after admission, then measured by laboratory of hospital. Demographic and blood examination information were collected by hospital medical records, including age, sex, blood pressure, blood routine, inflammation, coagulation and lipid related indicators. NLR was calculated as the ration of neutrophils divided by lymphocytes.

### Statistical analysis

Continuous variable was expressed as mean ± SD, median and interquartile range according to its distribution. One-way ANOVA or Kruskal–Wallis H test were used to examine the differences among groups. Categorical variable was shown as percentage, and the Chi-square or Fisher’s exact test was used to examine the differences. Multiple logistic regression analysis was preformed to examine associations with outcome, controlling for potential confounders. Receiver operating characteristic curve analysis was performed to determine the diagnostic performance of NLR and FIB in three groups. Statistical analyses were performed using IBM SPSS (version 26; SPSS Inc, Chicago, Ill) and GraphPad Prism. The reported P-value was two-tailed, and P < 0.05 was considered to be statistically significant.

## Results

Clinical and blood biochemistry indicators in the three groups were listed in Table 1.There was no significant difference in age, sex among the groups. Hypertension, the most frequency risk factors for dissection/aneurysm, didn’t differ between the three groups (P = 0.319). WBC count, PLR, D-dimer and FDP were higher in dissection group than the other two groups. Cholesterol, LDL, Eosinophil (EO), BNP and CKMB were little higher in aneurysm group.

**Table 1:** Basic and laboratory date among the three group.

	Dissection group (n = 606)	Aneurysm group (n = 202)	Healthy group (n = 140)	*P* value
Age (year)	59.88 ± 13.05	60.47 ± 17.72	57.44 ± 11.04	0.112
Male	485 (80)	164 (81.19)	113 (80.71)	0.932
Hypertension	449 (74.1)	140 (69.3)	100 (71.4)	0.391
Cholesterol (mmol/l)	4.42 (3.82–4.98)	4.52 (3.83–5.31)	4.42 (3.82–4.97)	0.040
Triglyceride (mmol/l)	1.27 (0.93–1.73)	1.36 (0.92–1.93)	1.30 (0.92–1.7)	0.221
HDL (mmol/l)	1.16 (0.96–1.36)	1.16 (1.01–1.22)	1.22 (1.04–1.42)	0.035
LDL (mmol/l)	2.54 (2.09–3.02)	2.78 (2.31–2.97)	2.64 (2.15–3.09)	0.011
WBC (× 10^9^/l)	8.05 (6.25–10.81)	6.37 (5.28–7.92)	6.07 (5.12–7.21)	< 0.001
NLR (mg/l)	4.52 (2.7–8.13)	2.43 (1.75–3.94)	2.03 (1.57–2.49)	< 0.001
PLR (mg/l)	149.77 (109.82–209.89)	120.19 (91.78–160.71)	112.9 (87.94–140.6)	< 0.001
EO (× 10^9^/l)	0.07 (0.01–0.16)	0.14 (0.06–0.22)	0.11 (0.07–0.18)	< 0.001
d-Dimer (mg/ml)	2.12 (0.82–3.95)	1.66 (0.5–4.35)	0.27 (0.22–0.32)	< 0.001
FIB (g/ml)	4.03 (3.1–5.4)	3.44 (2.9–4.11)	2.97 (2.58–3.39)	< 0.001
FDP (ug/ml)	8.09 (3.54–16.26)	5.74 (2.49–15.06)	1.99 (1.7–2.27)	< 0.001
APTT (s)	38.6 (35–42.5)	38.15 (34.7–41.03)	35.85 (33.9–38.55)	< 0.001
BNP (pg/ml)	66.29 (4.09–202.81)	101.66 (26.79–207.22)	23.6 (11.38–32.04)	0.013
CKMB (ng/ml)	3.65 (0.8–10.74)	5.08 (1.1–9.68)	1.73 (1–2.76)	0.045

*HDL* High density lipoprotein, *LDL* Low density lipoprotein, *WBC* White blood cell, *NLR* Neutrophil/lymphocyte ratio, *PLR* Platelet/lymphocyte ratio, *EO* Eosinophil, *FIB* Fibrinogen, *FDP* Fibrinogen degradation product, *APTT* Activated partial thromboplastin time, *BNP* Brain natriuretic peptide, *CKMB* Creatine kinase isoenzyme MB.

**Figure 2:**
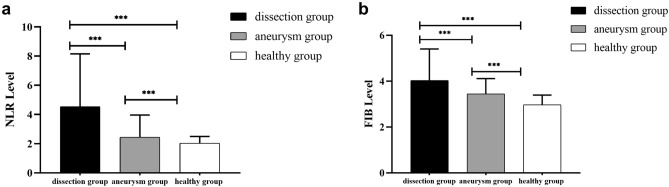
The NLR and FIB levels among three groups. (**a**) NLR levels; (**b**) FIB levels. ***P < 0.001.

The level of NLR in dissection group [4.52 (2.7–8.13)] was significantly higher than aneurysm group [2.43 (1.75–3.94)] and healthy group [2.03 (1.57–2.49)] [P < 0.001, Fig. 2a]. The FIB level in dissection group [4.03 (3.1–5.4)] was also significantly higher than that both in aneurysm group [3.44 (2.9–4.11)] and healthy group [2.97 (2.58–3.39)] [P < 0.001, Fig. 2b].

**Figure 3:**
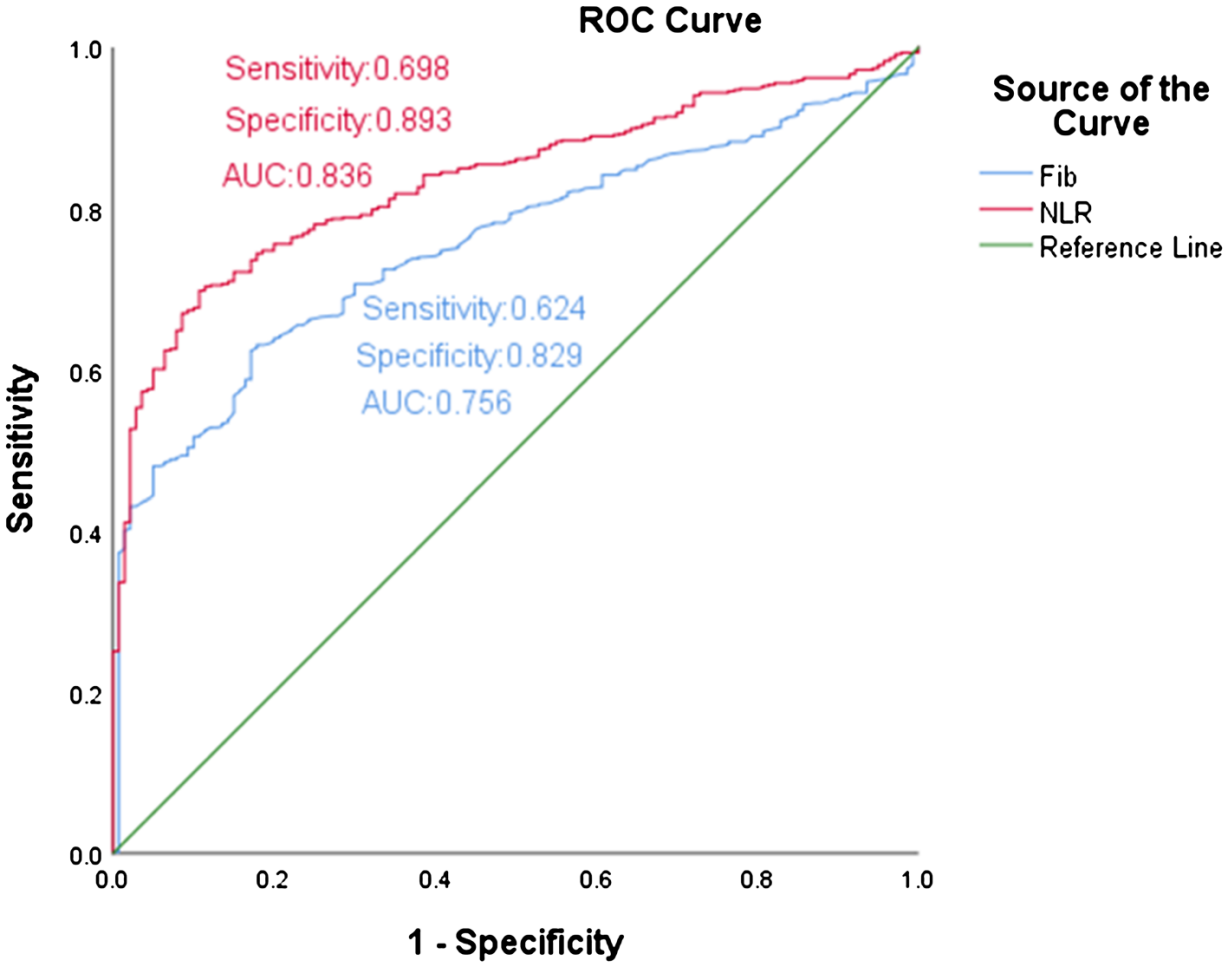
The ROC curves of NLR and FIB to distinguish TBAD patients.

According to univariate and multivariate logistic regression analysis, NLR and FIB were independent risk factors of the dissection group, and the OR and 95% CI value of NLR and FIB were 1.499 (1.126–1.738) and 1.914 (1.475–2.485), respectively (Table 2). ROC analysis was performed to evaluate the diagnostic implication of NLR and FIB in dissection group. The area under the curve (AUC) was 0.836 of NLR and 0.756 of FIB. NLR and FIB showed high specificity, 89% and 83% respectively (Fig. 3).

**Table 2:** Univariate and multivariate analysis among the three group.

	Univariate		Multivariate	
	OR and 95% CI	*P* value	OR and 95% CI	*P* value
Cholesterol (mmol/l)	1.147 (0.955–1.377)	0.143	NA	NA
HDL (mmol/l)	0.607 (0.361–1.021)	0.06	NA	NA
LDL (mmol/l)	0.991 (0.781–1.258)	0.941	NA	NA
WBC (× 10^9^/l)	1.408 (1.285–1.543)	< 0.001	1.096 (0.953–1.260)	0.197
NLR (mg/l)	2.013 (1.695–2.392)	< 0.001	1.499 (1.126–1.738)	< 0.001
PLR (mg/l)	1.013 (1.009–1.016)	< 0.001	1.004 (0.998–1.009)	0.182
EO (× 10^9^/l)	0.462 (0.131–1.634)	0.231	NA	NA
d-Dimer (mg/ml)	1.941 (1.23–1.892)	< 0.000	1.276 (1.034–1.133)	0.005
FIB (g/ml)	2.115 (1.753–2.553)	< 0.001	1.914 (1.475–2.485)	< 0.001
FDP (ug/ml)	1.348 (1.257–1.446)	< 0.001	1.215 (1.207–1.432)	0.008
APTT (s)	1.094 (1.055–1.134)	< 0.001	0.998 (0.950–1.049)	0.994
BNP (pg/ml)	1.001 (1–1.002)	0.001	1.0 (0.99–1.001)	0.785
CKMB (ng/ml)	1.017 (1.003–1.031)	0.02	1.048 (1.014–1.083)	0.015

NA represented non data available.

## Discussion

This study found that preoperative NLR and FIB were higher in patients with TBAD compared to both aortic aneurysm and healthy participants. NLR and FIB showed excellent specificity, which was different from previous published diagnostic biomarkers. Just as we suspected NLR and FIB maybe a powerful indicator in improving diagnostic accuracy for TBAD.

Biomarkers were widely used in cardiovascular diseases because of their low cost, easy availability and less waiting time. Previous studies presented that D-dimer had excellent sensitivity but modest specificity diagnostic performance. Troponin T can be used for early risk stratification of patients with acute type A aortic dissection^25^. White blood cells and N-terminal pro-brain natriuretic peptide were suggested for early diagnosis of aortic dissection^26^. In this study, NLR and FIB showed wonderful specificity, that maybe a powerful complementary with the existing biomarkers to improve the diagnostic value.

Compared with neutrophil count, lymphocyte count, NLR is more stable in the prediction of aortic disease. Studies have shown that there is a significant correlation between NLR and systemic inflammation^27^. Accumulating evidence has shown the value of NLR in evaluating the prognosis of aortic diseases^28–31^. Elevated NLR is also positively associated with the development of hypertension, stroke, and acute myocardial infarction (AMI)^32–34^. Aurelian et al*.* found that NLR is significantly higher among patients with infrarenal abdominal aortic aneurysm (rAAA) and that an NLR > 5 indicates a 5 times greater possibility of AAA being ruptured^35^. However, the prognostic capacity of NLR on admission is yet to be clarified in patients with type B AAD. Fibrinogen is associated with preoperative hypoxemia in patients with aortic dissection^36^. Low preoperative fibrinogen level is a risk factor for neurological complications and in-hospital mortality in patients with acute aortic dissection^37,38^. In addition, studies have shown that fibrinogen-fibrin degradation product level at admission can be used as a predictor of aortic growth and poor 1-year outcome in uncomplicated type B aortic dissection^39,40^. This is the first study that evaluated the potential diagnosis performance of FIB in patients with TBAD.

The function and mechanism of NLR in TBAD morbidity are still unclear. Elevated neutrophils interaction with endothelial cells leading to vascular intima injury. In addition, inflammatory cells can increase matrix metalloproteinases, which can degrade collagen and elastin, resulting in structural destruction of the aortic wall, resulting in aortic dissection^41,42^. Fibrinogen is an important protein in the process of coagulation and hemostasis. It can be abnormally elevated during infection, trauma, inflammation, surgery or tumor as a direct coagulation factor involved in the coagulation process. The coagulation disorder of patients with acute aortic dissection can be improved by further increasing the level of fibrinogen before operation^43^. When vascular endothelial cells are damaged in patients with aortic dissection, the increase of fibrinogen can be caused by endogenous and exogenous coagulation pathways^44^. This study shows that the level of fibrinogen in patients with aortic dissection is significantly higher than that in other groups, suggesting that the coagulation function of patients with aortic dissection is enhanced, which is helpful for pseudocavitary thromboembolization^45^.

This study is the first to combine NLR and FIB in the diagnosis of type B aortic dissection, which has a high clinical application value. However, there are still several disadvantages in this study: (1) the sample size of patients with non-aortic dissection is small; (2) This study is retrospective observational study; (3) Further studies are needed to understand the diagnostic role of NLR and FIB in type B aortic dissection, including combined use with other biomarkers; (4) Since many inflammatory and malignant diseases other than AD were associated with the research parameters, the patients in the study without excluding co-founder diseases.

## Conclusion

NLR levels are significantly higher among patients with Type B aortic dissection than those in abdominal aneurysm and healthy controls. Elevated NLR levels may contribute to the pathogenesis of aortic dissection. Further large clinical studies to investigate whether NLR levels are an independently risk factor for aortic dissection are warranted.

## Abbreviations

AAD: Acute aortic dissection
AMI: Acute myocardial infarction
APE: Acute pulmonary embolism
APTT: Activated partial thromboplastin time
BNP: Brain Natriuretic Peptide
CT: Computed tomography
CTA: Computed tomography angiography
CKMB: Creatine kinase isoenzyme MB
DSA: Digital subtraction angiography
EO: Eosinophil
FDP: Fibrinogen degradation product
FIB: Fibrinogen
HDL: High-density lipoprotein
INR: International normalized ratio
LDL: Low-density lipoprotein
Lymp: Lymphocyte
NLR: Neutrophil to lymphocyte ratio
PLT: Platelet
WBC: White blood cell
